# Suppression and resolution of autoimmune arthritis by rhesus θ-defensin-1, an immunomodulatory macrocyclic peptide

**DOI:** 10.1371/journal.pone.0187868

**Published:** 2017-11-16

**Authors:** Justin B. Schaal, Dat Q. Tran, Akshay Subramanian, Reshma Patel, Teresina Laragione, Kevin D. Roberts, Katie Trinh, Prasad Tongaonkar, Patti A. Tran, Dmitriy Minond, Gregg B. Fields, Paul Beringer, André J. Ouellette, Percio S. Gulko, Michael E. Selsted

**Affiliations:** 1 Department of Pathology & Laboratory Medicine, Keck School of Medicine, University of Southern California, Los Angeles, California, United States of America; 2 Oryn Therapeutics, Vacaville, California, United States of America; 3 Children’s Hospital Los Angeles, Los Angeles, California, United States of America; 4 Division of Rheumatology, Department of Medicine, Icahn School of Medicine at Mount Sinai, New York, New York, United States of America; 5 Torrey Pines Institute for Molecular Studies, Port St Lucie, Florida, United States of America; 6 Department of Chemistry & Biochemistry, Florida Atlantic University, Jupiter, Florida, United States of America; 7 The Scripps Research Institute, Jupiter, Florida, United States of America; 8 School of Pharmacy, University of Southern California, Los Angeles, California, United States of America; 9 Norris Comprehensive Cancer Center of the University of Southern California, Los Angeles, California, United States of America; Public Library of Science, FRANCE

## Abstract

θ-defensins constitute a family of macrocyclic peptides expressed exclusively in Old World monkeys. The peptides are pleiotropic effectors of innate immunity, possessing broad spectrum antimicrobial activities and immunoregulatory properties. Here we report that rhesus θ-defensin 1 (RTD-1) is highly effective in arresting and reversing joint disease in a rodent model of rheumatoid arthritis (RA). Parenteral RTD-1 treatment of DA/OlaHsd rats with established pristane-induced arthritis (PIA) rapidly suppressed joint disease progression, restored limb mobility, and preserved normal joint architecture. RTD-1 significantly reduced joint IL-1β levels compared with controls. RTD-1 dose-dependently inhibited fibroblast-like synoviocyte (FLS) invasiveness and FLS IL-6 production. Consistent with the inhibition of FLS invasiveness, RTD-1 was a potent inhibitor of arthritogenic proteases including ADAMs 17 and 10 which activate TNFα, and inhibited matrix metalloproteases, and cathepsin K. RTD-1 was non-toxic, non-immunogenic, and effective when administered as infrequently as once every five days. Thus θ-defensins, which are absent in humans, have potential as retroevolutionary biologics for the treatment of RA.

## Introduction

Rheumatoid arthritis (RA) is a systemic autoimmune disease that affects 0.5 to 1% the world population [1]. RA is typically characterized by symmetric polyarthritis involving the extremities with erosive joint changes mediated by hyperplastic synovium (pannus) and chronic inflammation [1]. Disease severity is associated with elevated production of inflammatory cytokines and activities of tissue degrading proteases [1]. While the etiology of RA is incompletely understood, studies in rodent RA models have provided major insights into the pathophysiology of RA including the discovery of dysregulation of cytokine signaling networks, providing bases for the development of new treatments. Central roles of TNFα, IL-1β, and IL-6 [2, 3] have provided rationale for therapies targeting these arthritogenic cytokines. While these and other interventions have greatly improved disease control and the quality of life of RA patients, the majority of patients achieve moderate and incomplete disease control, and remission is achieved in only 10–15% of patients [4]. Side effects of approved RA therapeutics have limited their use in a number of patient populations [5]. Thus, new and more efficacious therapies are needed for RA and major efforts are underway to identify and develop novel treatment strategies.

**Fig 1:**
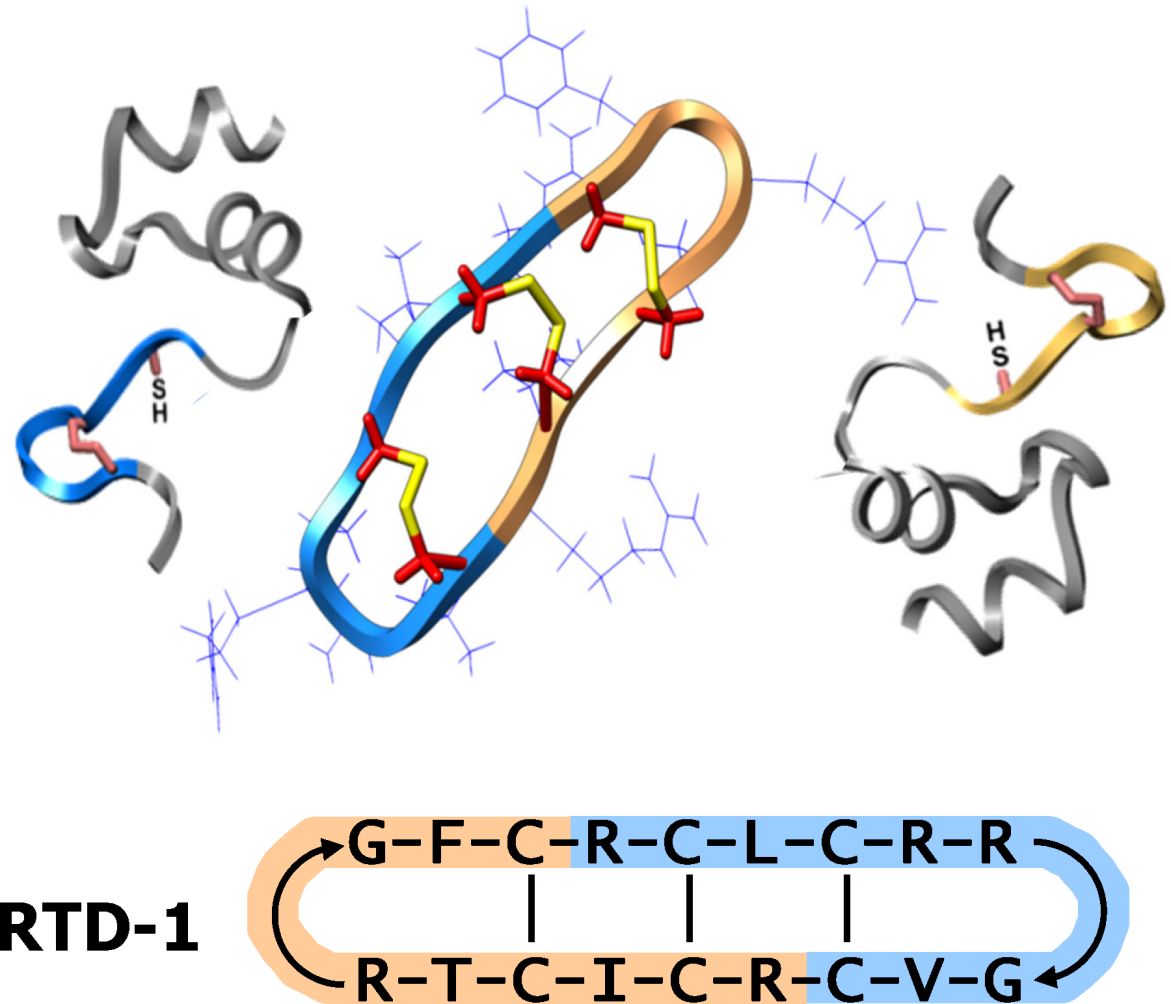
Biosynthesis and structural features of θ-defensins. RTD-1 is an 18-amino acid, tridisulfide, macrocyclic peptide produced by head-to-tail splicing of two 9-amino acid oligopeptides (color-coded) that are excised from corresponding propeptides [8, 13]. A schematic of RTD-1 with amino acid sequence and disulfide connectivities is shown.

Defensins are host defense peptides, composed of three evolutionarily related structural families (designated as α-, β-, and θ-defensins) that function as antimicrobial effectors of innate immunity [6, 7]. While most mammals express α- and β-defensins, θ-defensins are unique to Old World monkeys, and are absent in other primates including humans and other hominids [6]. θ-defensins are macrocyclic, tridisulfide-stabilized 18-amino acid molecules produced by post-translational head-to-tail splicing of two nonapeptides (Fig 1) [8]. They are the only known cyclic polypeptides in animals, and their conformation confers remarkable stability *in vitro* and *in vivo* [9, 10]. Six and ten θ-defensin isoforms are produced in rhesus monkeys [11] and baboons [12], respectively.

RTD-1 (Fig 1) is the prototype θ-defensin and has therapeutic efficacy in infectious disease models. The peptide was highly effective in reducing lethality in mice with experimental severe acute respiratory syndrome (SARS) [14] and in murine models of *E*. *coli* and polymicrobial sepsis [9]. Therapeutic effects of RTD-1 were mediated, at least in part, by modulation of pro-inflammatory cytokines. *In vitro* studies revealed that RTDs suppress expression and secretion of pro-inflammatory cytokines including TNFα, IL-1β, and IL-6 [9, 14] by TLR agonist-stimulated leukocytes, and the anti-inflammatory effects are mediated by regulation of NF-κB and MAPK signaling pathways [15]. These studies suggest that θ-defensins are pleiotropic peptides that evolved to neutralize pathogens and modulate inflammation. To further test this concept, we evaluated the effect of RTD-1 on the course of pristane-induced arthritis (PIA) in DA rats, a model of autoimmune arthritis that shares many features of human RA [16]. Based on the finding that RTD-1 was effective in dose-dependently suppressing and resolving PIA, we conducted studies to identify mechanisms that may mediate the therapeutic efficacy observed.

## Materials and methods

### Ethics

Animal use was approved by the University of Southern California (USC) Institutional Animal Use and Care Committee (protocols 11355 and 20378). Sequential blood sampling for pharmacokinetic analyses was performed by Bioanalytical Systems, Inc. (BASi, West Lafayette, IN), an AAALAC-accredited facility. All animal studies were in compliance with the National Institutes of Health Guide for the Care and Use of Laboratory Animals. Animals were euthanized by carbon dioxide inhalation with confirmation by thoracotomy per IACUC guidelines.

### Anti-arthritic agents

Methotrexate (MTX; Sigma-Aldrich, St. Louis, MO) was prepared in saline. Etanercept (Enbrel™; Immunex Corp., Thousand Oaks, CA) was obtained from the USC Investigational Drug Service Pharmacy and was reconstituted per manufacturer’s instructions.

### Peptides

The hydrochloride salt of RTD-1 was prepared as previously reported [8, 13] producing highly pure (> 98%) material as assessed by analytical reverse-phase ultra-performance liquid chromatography (RP-UPLC) and tandem mass spectrometry. Sterile stock solutions of RTD-1 were dissolved in normal saline and stored at 4–8°C.

### Pristane-induced arthritis (PIA)

Female DA/OlaHsd (D blood group allele and Agouti, also known as Dark Agouti), rats were obtained from Harlan Laboratories (Indianapolis, IN). Animals were allowed to acclimate for 48–72 hours prior to experimental use. Animals were fed standard chow *ad lib* during a 12-hour day/night cycle. PIA was induced in 5–7 week old animals (90–100 gm) by intradermal injection of 250 μl pristane (Sigma) divided into 3–5 sites at the base of the tail [17]. Animals were monitored daily and disease severity of each limb was scored 0–4 using the method of Brand *et al*. [18]. The severity scores for all limbs of individual animals were totaled to give an individual’s arthritis severity score (AS) ranging from zero for normal animals to maximum disease score of sixteen. Disease severity was assessed by at least two scorers and the inter-rater agreement (kappa) value was 0.806 as determined using MedCalc Statistical Software version 14.8.1 (MedCalc Software, Ostend, Belgium). In three separate trials, treatment was also blinded to scorers. There was no statistical difference between blinded and unblinded scoring of any treatment cohort. Treatment with saline (vehicle), RTD-1, or anti-arthritic agents was randomized and initiated when an animal first presented with an AS of 3 or more. RTD-1 was administered subcutaneously with doses ranging from 0.2 to 5.0 mg/kg in an injection volume of 0.5 ml at intervals ranging from once a day (qd) to once every seven days (q7d). MTX was administered at 0.25 mg/kg by intraperitoneal injection q3d [19]. Etanercept was administered at 0.4 mg/kg by subcutaneous injection q3d [20, 21].

### Joint histopathology

Limbs from euthanized animals were preserved in buffered formalin, decalcified, embedded, sectioned, and stained with hematoxylin and eosin (H & E) in the USC Research Model Pathology Core. Microscopic images were acquired with a Nikon-Microphot FXA with SPOT 5.1 image capture software.

### RTD-1 pharmacokinetics

Eight to 12-week old male Sprague Dawley rats (Harlan) were fitted with a surgically implanted jugular vein catheter for sequential blood collection (Culex NxT Automated In Vivo Sampling System; BASi, West Lafayette, IN), and monitored daily. RTD-1 dissolved in saline was injected subcutaneously at 5 mg/kg to 4 animals. Approximately 200 μl of blood was collected before peptide administration and at 0.25, 0.5, 1, 2, 4, 6, 8, 12, 18, 24, 30, 36, 48, 60, and 72 h after RTD-1 injection. Blood was collected into vials containing K_3_EDTA and plasma was collected after centrifugation. Plasma concentrations of RTD-1 were determined after solid phase extraction (SPE) by weak cation exchange (Oasis WCX, Waters, Milford, MA) and quantitation by C18 RP-UPLC, photodiode array (PDA) detection, and electrospray-ionization mass spectrometry (ESI-MS). Chromatography was performed on an Acquity H-class UPLC with an analytical PDA detector using Empower 3 software (Waters). Quantitative mass spectrometry was performed on post PDA eluent using a Micromass Quattro Ultima mass spectrometer with MassLynx 4.1 (Waters). Pharmacokinetic analysis was performed using parametric population modeling with maximum likelihood estimation via the EM algorithm with sampling (MLEM) as implemented in the ADAPT 5 (Version 5.0.49) PK/PD Systems Analysis Software (Biomedical Simulations Resource, University of Southern California).

### Joint tissue cytokine analysis

Saline (control) and RTD-1 (5 mg/kg, s.c., qd) treated PIA rats were euthanized 4 days after disease onset (AS ≥ 3). The ankle joints and feet were snap frozen in liquid N_2_ and pulverized with mortar and pestle as described [22]. One gram of tissue powder was extracted with 5 ml of ice cold 10 mM sodium phosphate, 150 mM NaCl, pH 7.4 (PBS) containing protease inhibitor tablets (cOmplete ULTRA with EDTA; Roche Diagnostics GmbH, Mannheim, Germany). Samples were vortexed for 30 seconds and extracts clarified by centrifugation. Supernatant IL-1β was quantified by ELISA (Life Technologies, Grand Island, NY).

### Isolation of FLS from DA rats

Fibroblast-like synoviocytes (FLS) were isolated by enzymatic digestion of the synovial tissue from DA rats with PIA as previously described [23]. Cells were washed and suspended in DMEM supplemented with 10% fetal bovine serum (Gibco, Grand Island, NY), 30 mg/ml L-glutamine (Sigma), 250 μg/ml amphotericin B (Sigma), and 10 mg/ml gentamicin (Gibco). After overnight culture, non-adherent cells were removed and adherent cells were cultured to approximately 70–90% confluence and passaged following detachment with 0.25% trypsin-EDTA for 3 min at 37°C. All experiments were performed with FLS after at least four passages.

### Invasion assay

FLS invasiveness was assayed *in vitro* using Matrigel-coated inserts in a transwell system (Becton, Dickinson and Company, Franklin Lakes, NJ) as previously described [23]. Briefly, DA rat FLS (70–80% confluent) isolated as described above were suspended at 2.0 × 10^4^ cells/well in serum-free DMEM. Cells were placed in the upper compartment of the Matrigel-coated inserts to which RTD-1 was added to final concentrations of 0.1, 1, or 10 μg/ml. The lower compartment contained complete medium with 10% FBS and plates were incubated at 37°C. After 24 hours the upper surface of the insert was wiped with cotton swabs to remove non-invading cells and the Matrigel layer. The opposite side of the insert was stained with Crystal Violet (Sigma) and the total number of invading cells was counted at 100X magnification. Experiments were performed in duplicate on seven cell lines each generated from the synovial tissue of individual DA rats with PIA.

### Expression of IL-6 by stimulated FLS

FLS were cultured in 12-well plates at 4–6 × 10^4^ cells/well for 24 h (viability > 95% by trypan blue staining). Medium was replaced and FLS were incubated with TNFα or IL-1β at final concentrations of 3 or 10 ng/ml plus RTD-1 (10 μg/ml in 0.01% acetic acid (HOAc)) or vehicle alone. After 24 h incubation, FLS culture supernatants were removed, clarified by centrifugation, and analyzed for IL-6 by ELISA (Invitrogen, Carlsbad, CA).

### FLS proliferation

FLS were seeded at 3 × 10^3^ cells/well in a 96-well TC treated plate (Greiner). Cells were allowed to adhere for 18 h and medium was replaced prior to addition of peptide or vehicle. RTD-1 was added to wells at final concentrations of 0–30 μg/ml in culture medium and incubated for 0, 24, or 48 h at 37°C in 5% CO_2_. FLS cell number was determined by DNA staining using CyQuant Cell Proliferation Assay Kit (Invitrogen) per manufacturer’s instructions. Cellular toxicity was determined by measuring the release of lactate dehydrogenase (LDH) using a CytoTox 96 Cytotoxicity Assay (Promega Bio Sciences, San Luis Obispo, CA) in FLS supernatants. All assays were performed with ≥ 7 replicates.

### Protease inhibition assays

RTD-1 was analyzed for enzymatic inhibition against zinc metalloproteases ADAM17, ADAM10, matrix metalloproteases (MMPs) 1, 2, 3, 8, 9, 13, and 14, and cysteine cathepsins (Cats) B, C, H, K, L, S, and V (Table 1). ADAM10 and ADAM17 activities were measured by release of fluorogenic peptides cleaved from pro-TNF (R&D Systems, Minneapolis, MN). ADAM10 was diluted to 0.05 μg/ml and ADAM17 to 0.1 μg/ml with 25 mM TRIS, 2.5 μM ZnCl_2_, 0.005% Brij35, pH 9.0 and incubated with 0 to 3 μg/ml RTD-1 dissolved in 0.01% HOAc. Assay was initiated by addition of substrate (10 μM final) and enzymatic activity measured at 37°C (ADAM10) or 22°C (ADAM17) every 30 seconds for 1 h in a SpectraMax M5e fluorometer (Molecular Devices, Sunnyvale, CA) with 320 nm excitation and 405 nm emission.

**Table 1:** RTD-1 inhibition of proteinases associated with RA pathogenesis.

Enzyme	IC_50_ (μM)	Substrate (10 μM)	^a^ EC (nM)	Enzyme substrates implicated in RA
ADAM 10	0.45 ± 0.2	Mca-KPLGL-Dpa-AR-NH_2_	0.96	ProTNF, Fas ligand, IL-1R, LAG-3	
ADAM 17	0.11 ± 0.04	Mca-PLAQAV-Dpa-RSSSR-NH_2_	1.9	ProTNF, Fas ligand, TNFRs, IL-1R, IL-6R, L-selectin, LAG-3, RANKL	
Cathepsin B	1.0 ± 0.1	z-LR-AMC	1.5	Proteoglycans, aggrecan, fibronectin, laminin, facit, osteocalcin, osteonectin, collagen IV	
Cathepsin C	3.7 ± 0.6	GL-AMC	9.8	Activation of granule serine peptidases	
Cathepsin H	>50	R-AMC	36	Osteocalcin	
Cathepsin K	0.027 ± 0.02	z-FR-AMC	0.77	Collagens I, II, aggregans, elastin, osteonectin	
Cathepsin L	1.4 ± 0.3	z-LR-AMC	0.39	Proteoglycans, aggrecan, basement membrane type IV, elastin, fibronectin, laminin, osteocalcin	
Cathepsin S	>50	Mca-RPKPVE-Nval-WRK(Dnp)-NH_2_	27	Collagens, elastin, fibronectin, osteocalcin	
Cathepsin V	1.8 ± 0.6	z-LR-AMC	6.8	Elastin	
MMP-1	>50	Mca-PLGL-Dpa-AR-NH_2_	2.0	Collagens I, II, III	
MMP-2	19.6 ± 0.04	Mca-PLGL-Dpa-AR-NH_2_	2.0	Collagen IV	
MMP-3	>50	Mca-RPKPVE-Nval-WRK(Dnp)-NH_2_	48	Collagens IV, IX, and X, proteoglycans, fibronectin, laminin, elastin	
MMP-8	12.0 ± 0.05	Mca-PLGL-Dpa-AR-NH_2_	2.0	Collagens I, II, III	
MMP-9	11.1 ± 0.2	Mca-PLGL-Dpa-AR-NH_2_	2.0	Collagens IV and V	
MMP-13	11.0 ± 0.4	Mca-PLGL-Dpa-AR-NH_2_	2.0	Collagens I, II, III	
MMP-14	7.8 ± 0.3	Mca-PLGL-Dpa-AR-NH_2_	2.0	Collagens I, II, III, proMMP-2	

^a^ EC–enzyme concentration; see Methods for enzyme assay conditions.

RTD-1 inhibition of Cat B (BPS Bioscience, San Diego, CA) and Cats C, H, K, L, S, and V (R & D Systems) was analyzed in a 96 well format with enzyme-specific substrates per manufacturer’s instructions and as described in Table 1. The activity of each enzyme was determined in the presence of 0 to 50 μg/ml RTD-1.

Inhibition of MMPs by RTD-1 was performed as described [24], incubating each enzyme with 0–100 μg/ml of RTD-1. IC_50_ values were calculated by GraphPad Prism 5.01 (GraphPad Software, San Diego, CA) utilizing a non-linear curve fit of log [inhibitor] versus response with variable slope.

### RTD-1 immunogenicity analysis

Adult DA or Sprague-Dawley rats were challenged s.c. with 5 mg/kg RTD-1 every day (15–42 injections), or every other day (8 injections). Several animals were boosted 2 months later, prior to serum or plasma collection. Immune responses were analyzed by dot blot on nitrocellulose membranes as described in legend to the figure.

### Statistics

Statistical analyses were performed utilizing GraphPad Prism 5.01. All sample populations were tested for normality using a D’Agostino and Pearson omnibus normality test. P-values were generated in parametric sample means by student’s t-test while non-parametric sample medians were compared by Mann-Whitney U-test. Number of replicates and/or total number of animals are shown in figure legends or within the figures.

## Results

### Efficacy of RTD-1 in rat PIA

**Fig 2:**
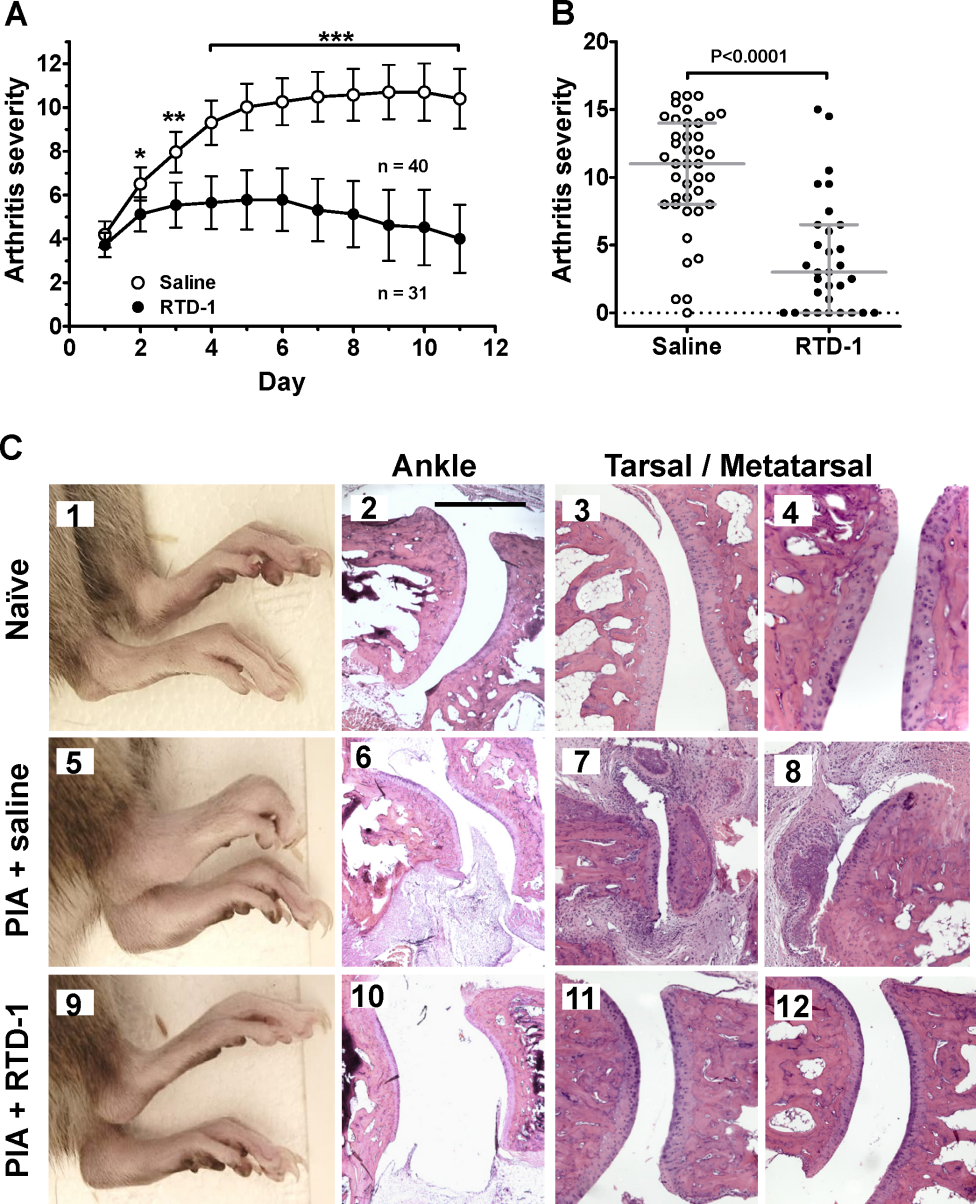
Efficacy of RTD-1 in rat PIA. A. DA rats with established PIA were treated s.c. daily with RTD-1 (5 mg/kg) or vehicle (saline), with cohort randomization as described in Methods. RTD-1 treatment reduced arthritis severity scores within 24 h compared to control (* *P* = 0.0064), and therapeutic efficacy increased with duration of treatment (** *P* < 0.001; *** *P* < 0.0001, mean ± 95% CI, results of 6 independent experiments with n = 5–10 animals per treatment group per experiment). The larger number of saline-treated animals represented in the figure is a result of variation in randomization sequences across six experimental trials. B. Median AS (with interquartile ranges) of individual animals on day 11 from panel A. C. Representative images (10X magnification, bar = 500 μm) show gross and histologic features of hind extremities from naïve, saline-treated PIA rats (daily s.c. x 11 days), and RTD-1 treated PIA rats (daily s.c. 3 mg/kg for 9 or 11 days; see S1 Fig for additional examples of normal, PIA, and RTD-1-treated joint histology). Joints from saline-treated PIA rats show synovial hyperplasia, invasive pannus, and disruption of joint architecture (panels 6–8). RTD-1 treatment of PIA rats resulted in marked resolution of paw swelling as well as preservation of a normal joint architecture (panels 9–12).

The anti-arthritic effects of RTD-1 were tested in DA/OlaHsd rats with PIA, a well-characterized model of chronic RA [16, 22, 25–27]. Animals with evolving PIA (arthritis severity score, AS ≥ 3) were scored, randomly assigned to a treatment group, and treated daily with subcutaneous (s.c.) injections of saline or 5 mg/kg of RTD-1 for 11 days (Fig 2A). RTD-1 treatment significantly reduced arthritis progression within 24 h of the first administered dose (*P* = 0.0064; Fig 2A). RTD-1 treated rats had markedly lower (69% reduction of mean) AS at the end of the observation period compared with vehicle controls (*P* < 0.0001; Fig 2B). Additionally, complete resolution of disease (AS = 0) occurred in 29% (9 of 31) of RTD-1 treated animals compared to 2.5% (1 of 40) of saline treated animals (*P* = 2.9 × 10^−17^, Chi-squared) **(**Fig 2B). Further, RTD-1 treatment resulted in restoration of limb function and mobility (typical response shown in Supplemental S1 Video).

### Effect of RTD-1 on joint disease and histology in PIA rats

Joints from vehicle-treated PIA rats showed extensive swelling, synovial hyperplasia, invasive pannus, and erosion of cartilage and bone (Fig 2, panels 5–8; S1 Fig). In contrast, RTD-1 treatment markedly reduced erythema and swelling of the affected joints (Fig 2, panel 9) and dramatic improvement in function and weight bearing (S1 Video). The clinical improvement in RTD-1-treated rats correlated with preservation of normal joint histology (Fig 2; panels 10–12; S1 Fig) that was similar to limbs of naïve animals (Fig 2, panels 1–4, and S1 Fig), as joints from peptide-treated animals were devoid of synovial hyperplasia, inflammatory infiltration, angiogenesis, and erosive changes preserving a normal architecture.

### Efficacy of RTD-1 in severe PIA and comparison with RA drugs

**Fig 3:**
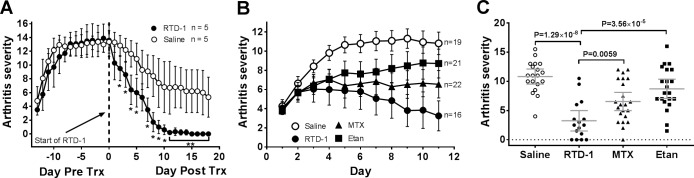
Efficacy of RTD-1 in severe PIA and comparison with RA drugs. A. RTD-1 induces resolution of advanced PIA (arthritis severity score, AS ≥ 12 prior to treatment) with significant (* *P* < 0.05; ** *P* < 0.005) reduction in AS (mean AS ± 95% CI) by day 2 of daily treatment with s.c. peptide at 5 mg/kg (results of two independent experiments). B. Efficacy of RTD-1 in rat PIA was superior to treatment with MTX or etanercept (Etan). All three agents significantly (*P* < 0.05) reduced arthritis progression by day 3 following treatment initiation, (mean AS ± 95% CI). C. Comparison of disease response to RTD-1, Etan, and MTX on day 11 (from panel B). RTD-1 treatment resulted in significantly greater disease resolution than MTX or Etan treatments. 0% (0 of 19) saline, 25% (4 of 16) RTD-1, 4.5% (1 of 22) MTX, and 0% (0 of 21) achieved complete resolution of disease (results of 2 independent experiments). Data are shown as individual animal AS and mean AS ± 95% CI is indicated with horizontal bars.

RTD-1 treatment was also effective in resolving advanced PIA. Rats with near maximum disease (AS ≥12) received daily s.c. injections of saline or 5 mg/kg of RTD-1, peptide treatment was continued until disease resolution (AS = 0) was achieved (6–9 RTD-1 treatments). Peptide treatment produced a rapid reduction in arthritis severity, which reached statistical significance within 48 hours of the start of treatment (*P* < 0.044), with complete resolution of clinical disease in all RTD-1 treated rats by day 15 (Fig 3A). As with animals treated in early PIA (Fig 2A), RTD-1 treatment of animals with severe PIA restored normal limb function and mobility (S2 Video). Of note, after a disease-free interval ranging from 4–12 weeks, three of five RTD-1 treated animals relapsed with joint disease (average AS score of ~3).

Efficacy of RTD-1 treatment was compared with etanercept (Etan) and methotrexate (MTX), first line RA drugs that have also been studied in rodent models of arthritis such as rat PIA [1, 19–22]. While both Etan and MTX limited the progression of arthritis by day 3 of treatment compared to vehicle controls (Fig 3B), treatment with RTD-1 had a more pronounced effect by treatment day 11 than either Etan or MTX, (*P* = 3.56 × 10^−5^, *P* = 0.0059, respectively). Additionally, RTD-1 achieved the highest rate of complete disease resolution (25%) compared to MTX (4.5%) or Etan (0%) (Fig 3C).

### Dose-dependent anti-arthritic effects and pharmacokinetics of RTD-1

**Fig 4:**
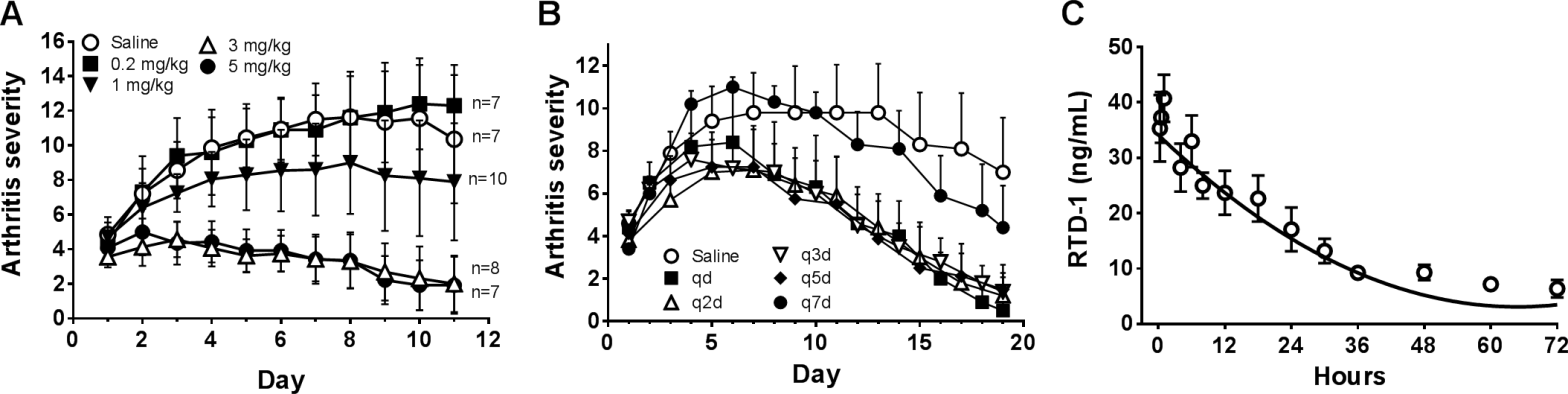
Dose-dependent anti-arthritic effects and pharmacokinetics of RTD-1. A. PIA rats receiving daily s.c. injection with 3 or 5 mg/kg of RTD-1 responded with significant (*P* ≤ 0.0015) and similar reductions in AS, compared to vehicle control, by treatment day 3. Dosing with 1 mg/kg RTD-1 resulted in modest, but not statistically significant reductions in AS, and dosing with 0.2 mg/kg produced no therapeutic effect (mean ± 95% CI, results of 2 independent experiments). B. S.c. injection of PIA rats with 5 mg/kg of RTD-1 daily, or once every 2, 3, or 5 days produced equivalent disease resolution (mean + 95% CI, n = 5 per treatment group, results of single experiment). AS were recorded every day and plotted every other day for clarity. C. Pharmacokinetics of s.c. administered RTD-1 was analyzed in Sprague Dawley rats (n = 4) that received a single 5 mg/kg s.c. dose of RTD-1. RTD-1 is rapidly distributed with clearance and half-life of 22.3 ml/kg/h and 0.285 h, respectively. Terminal clearance of RTD-1 was considerably slower at 3.55 ml/kg/hr (±0.286) with a terminal elimination half-life of 32.3 h (±10.4).

The anti-arthritic effects of RTD-1 in PIA were dose-dependent, with AS reductions observed with doses as low as 1 mg/kg (Fig 4A). Further, dosing of RTD-1 at 5 mg/kg was equally effective whether administered daily or once every 2, 3, or 5 days (Fig 4B). Analysis of single dose pharmacokinetics (PK) of s.c. administered RTD-1 revealed that the peptide disposition is best described by a 2-compartment model with relatively rapid distribution (clearance and half-life of 22.3 ml/kg/h and 0.285 h, respectively), but prolonged terminal elimination [RTD-1 clearance = 3.55 ml/kg/hr (±0.286)] and half-life of 32.3 h ±10.4 (Fig 4C). These PK parameters are consistent with the finding that RTD-1 is highly stable in human [9] and rat plasma and that q5d dosing is as effective as daily dosing (Fig 4B).

### RTD-1 is non-immunogenic

**Fig 5:**
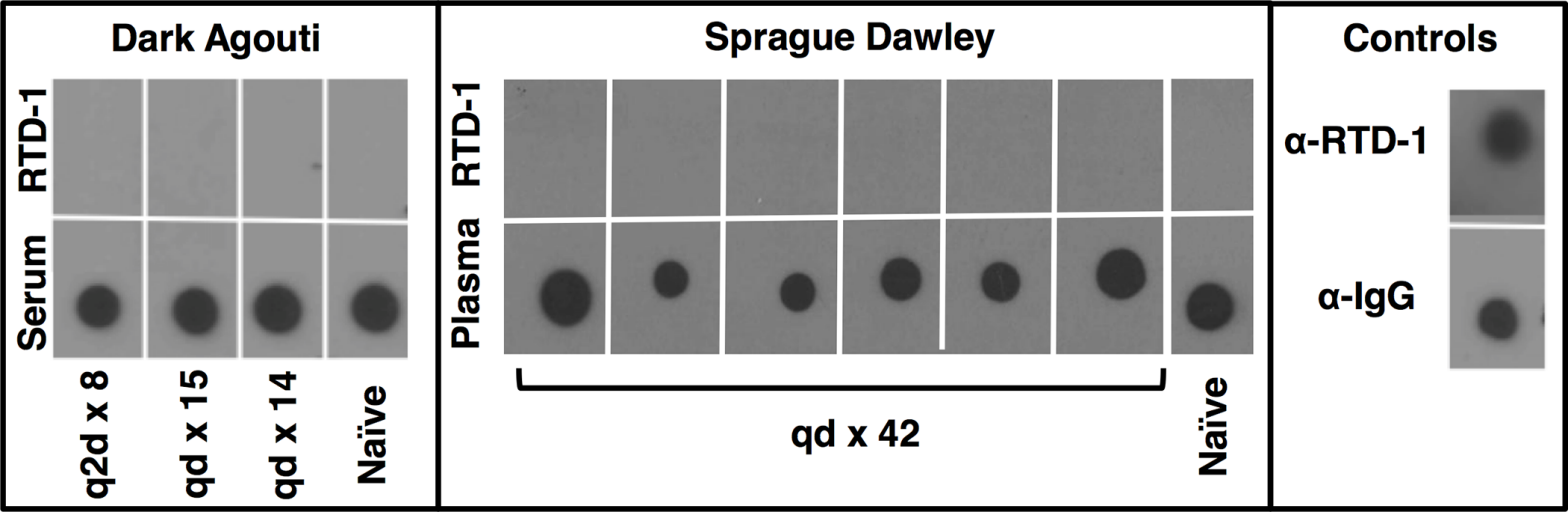
RTD-1 is non-immunogenic. DA rats were challenged 8–15 times and boosted prior to collection of serum, while Sprague Dawley rats were challenged daily for 42 consecutive days and plasma collected on day 43, as described in Methods. Serum or plasma was analyzed for presence of anti-RTD-1 IgG by dot blot. Right Panel: Dot blot controls for rat IgG and RTD-1 using horse anti-rat-IgG and goat anti-RTD-1, respectively. Left/center panels: Bottom row membranes were dotted with 1 μL of serum or plasma from rats challenged with RTD-1 as indicated, or from naïve (saline-only) controls. Bottom row membranes were probed with horse anti-rat-IgG (positive control). Top row membranes were dotted with 100 ng of RTD-1, and probed overnight with serum or plasma from either challenged or naïve rats. All membranes were developed with secondary antibodies (see Methods) and HRP detection.

To evaluate the immunogenicity of RTD-1, we challenged DA and Sprague Dawley rats with serial (8–42 daily) s.c. injections of 5 mg/kg RTD-1. Serum or plasma samples from animals were analyzed for humoral response by dot immunoblot assay. No anti-RTD-1 Ig was detected in any peptide-challenged animal (Fig 5), consistent with previous failed attempts to generate antibodies with unmodified RTD-1 [9].

### Effects of RTD-1 on arthritogenic cytokines

**Fig 6:**
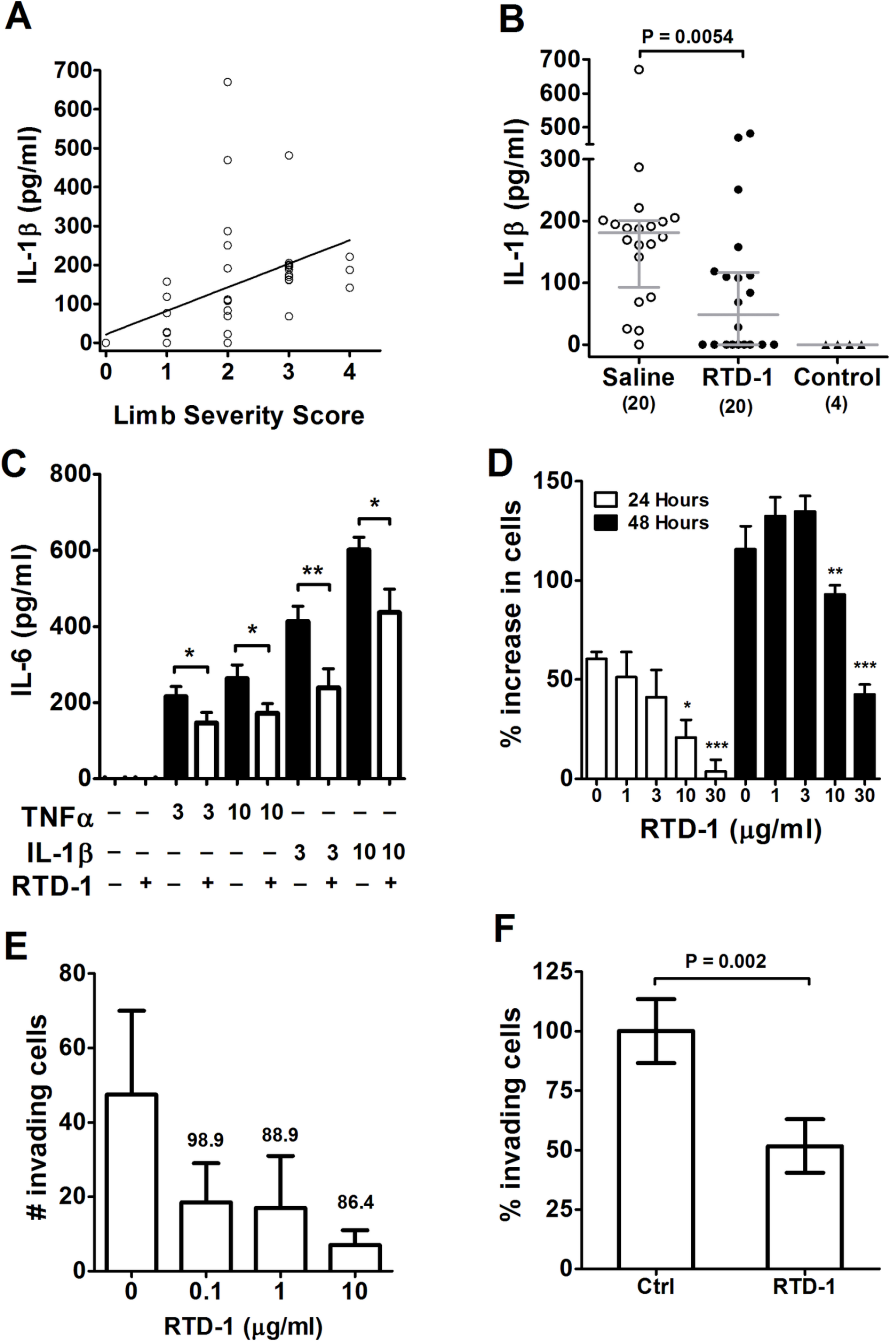
Effects of RTD-1 on arthritogenic mediators. A. IL-1β levels in joint homogenates increase with arthritis severity (n = 40 paw-ankle joints; Pearson r = 0.49, *P* = 0.0013). B. RTD-1 treatment reduced IL-1β levels in joints of PIA rats after 4 daily 5 mg/kg s.c. treatments compared to vehicle controls (brackets indicate number of paw-ankle joints). Data are shown as IL-1β levels in individual joint samples with medians with interquartile ranges indicated. C. RTD-1 (10 μg/ml) suppressed (3 or 10 ng/ml) TNFα- and IL-1β- stimulated production of IL-6 by cultured FLS (* *P* < 0.05, ** *P* = 0.0059) (mean + SE, n = 12; results of 3 independent experiments). D. RTD-1 dose-dependently suppressed proliferation of cultured DA-PIA-FLS. FLS cell numbers were quantified after 0, 24, and 48 h incubation with RTD-1 at the indicated concentrations (* *P* = 0.0023, ** *P* = 0.0243, *** *P* < 0.0001, *P*-values relate differences in cell proliferation relative to peptide free controls at corresponding time points) (mean + SE, n = 7, results of 2 independent experiments). E. RTD-1 suppressed invasion of FLS from PIA rats in a dose-dependent manner, with minimal cytotoxicity (percent viable cells above bars, n = 2). F. The anti-invasive effects of RTD-1 on 7 independently isolated rat FLS cell lines were analyzed (as in panel E). Incubation with 0.1 μg/ml RTD-1 inhibited invasion by 50% (mean ± 95% CI) obtained using 7 different rat DA cell lines.

IL-1β plays a central role in the pathogenesis of RA [28] and rodent arthritis [29–31]. In previous studies, we showed that RTD-1 suppresses IL-1β expression and release by immune-stimulated monocytes [9, 15]. We therefore analyzed the effect of RTD-1 administration on joint levels of IL-1β in PIA rats. Consistent with other studies, IL-1β was not detectable in joints of naïve rats, but levels were markedly elevated in diseased joints of PIA rats and joint IL-1β levels correlated with arthritis severity score of the corresponding limb (Pearson r = 0.4923, *P* = 0.0013; Fig 6A). PIA rats with established disease (AS ≥ 3) were treated s.c. once a day for four days with 5 mg/kg RTD-1 or vehicle after which joint tissue from euthanized animals was analyzed for IL-1β. Peptide treatment reduced joint IL-1β by an average of 44% (*P* = 0.0054, Fig 6B), which coincided with the marked reduction in arthritis severity in RTD-1 treated animals after 4 days of treatment (*P* = 0.0044; Fig 2A).

IL-6 is a pleiotropic cytokine, also implicated in RA pathogenesis by inducing differentiation of Th-17 cells, joint damaging proteases, angiogenesis, synoviocyte invasion [32, 33], and bone loss [34]. Stimulation of FLS from arthritic DA rats with TNFα or IL-1β dose-dependently induced IL-6 production, consistent with previous studies [35, 36]. RTD-1 significantly inhibited IL-6 production (27.3–42.2% reduction) by FLS (Fig 6C).

### Effects of RTD-1 on FLS proliferation and invasion

Arthritogenic cytokines stimulate the proliferation and invasion of FLS, and the *in vitro* invasive properties of these cells mediate and correlate with erosive joint changes in RA and PIA [23, 25, 37, 38]. Therefore, we analyzed the effect of RTD-1 on FLS proliferation and invasiveness. RTD-1 dose-dependently suppressed FLS proliferation when analyzed after 24 or 48 hours in culture (Fig 6D) and this effect was not a result of cytotoxicity as cell viability was ≥ 98% in all samples. RTD-1 also dose-dependently suppressed FLS invasion of Matrigel-coated inserts (Fig 6E). The invasion-suppressive effects were observed at low concentrations (100 ng/ml) and were highly reproducible against seven independent DA rat FLS cell lines (Fig 6F).

### Effects of RTD-1 on arthritogenic proteases

RTD-1 is a potent and extremely rapid inhibitor of TNFα release by LPS-stimulated leukocytes [9]. Based on the kinetics of these effects, we hypothesized that RTD-1 inhibits TNFα release by inhibition of TNFα converting enzyme (TACE; ADAM17) [39]. RTD-1 was found to be a potent inhibitor of ADAM17 as well as ADAM10, a related proTNF sheddase (Table 1). Additional studies, currently underway, disclose that RTD-1 rapidly and reversibly inhibits TACE (manuscript in preparation).

With the finding that RTD-1 inhibits ADAMs 17 and 10, both of which are zinc metalloproteinases, we tested for peptide effects on zinc matrix metalloproteinases (MMPs) implicated in cartilage degradation in RA. RTD-1 selectively inhibited pathogenic MMPs 2, 8, 9, 13, and 14, with IC_50_ values ranging from 2 to 20 μM (Table 1). In parallel we analyzed the effects of RTD-1 on cysteine proteases implicated in RA. Surprisingly, RTD-1 also inhibited cysteine cathepsins B, C, K, L, and V, with its greatest potency against cathepsin K (IC_50_ **~** 27 nM; Table 1). Cathepsin K is the protease primarily responsible for degradation of bone matrix by osteoclasts [40]. These data indicate that RTD-1 is a cross class anti-protease that inhibits TNFα activation and matrix degrading proteinases implicated in RA joint damage.

## Discussion

Despite advances in RA treatment over the past 15 years, there persists an unmet need for more widely effective therapies given the significant fraction of patients who are partial responders or who are ineligible candidates for existing drugs [4]. Previous studies disclosed that RTD-1 modulates inflammatory cytokine expression in rodent infectious disease models [9, 14] and in immune-stimulated human leukocytes [9, 15]. In those studies, arthritogenic cytokines (TNFα, IL-1β, and IL-6) were among those down-regulated by RTD-1. Based on these findings, we hypothesized that RTD-1 would alter the course of disease in a rodent model of RA wherein disease onset and progression are driven by TNFα, IL-1β, and IL-6. The studies reported here employed rat PIA since this T-cell dependent disease model mimics human RA, including symmetrical involvement of peripheral joints, destruction of cartilage and bone, dysregulated pro-inflammatory cytokines/chemokines, rheumatoid factor positivity, and its chronic course. PIA is dependent on MHC class II-restricted T-cells [26] and influenced by non-MHC genes, producing cellular and humoral autoimmunity [16, 25, 41]. Non-MHC genes that influence rat PIA arthritis severity include TNFα, IL-1β [42], IL-6, IL-17 [43], and CCL-2, and joint damaging MMPs [25, 44]. Rat PIA is increasingly utilized as a model for preclinical evaluation of human RA drug candidates [16].

Here we report that RTD-1, the prototype θ-defensin, rapidly arrests PIA in DA rats and peptide treatment resulted in high rates of disease resolution. Therapeutic effects were rapid, and dose level and dose interval dependent. RTD-1 induced disease resolution more effectively than a TNFα inhibitor or MTX, and at rates exceeding that reported using a mouse anti-TNFα antibody [45], or the < 10% remission rate observed in treated RA patients [46]. Moreover, arrest of disease progression and resolution of arthritis was obtained with RTD-1 treatment in early and in severe stages of PIA, and therapeutic responses correlated with marked reductions in limb swelling, and recovery of limb function and mobility. In our studies on the effect of RTD-1 on evolving disease, resolution of clinical arthritis corresponded to histologic joint morphology which disclosed that treated animals had essentially normal joint structures whereas those from control animals showed severe pannus formation, chronic inflammation, and erosion (Fig 2C and S1 Fig). In this study, we did not perform histologic evaluation of joints from RTD-1 treated animals with maximum established disease (Fig 3A). Of note, peptide treatment of severely arthritic animals induced a higher frequency of complete clinical resolution (Fig 3A; S2 Video) than occurred with treatment of animals with evolving disease (Fig 2B), and this may be of translational significance since treatment of RA is usually initiated once disease is well established. We hypothesize that this somewhat paradoxical finding reflects pleiotropic anti-arthritic RTD-1 mechanisms which differentially affect the pathophysiology of evolving and established severe disease. Studies are underway to identify pathologic and/or resolution-promoting processes modulated by RTD-1 at different stages of PIA. Evaluation of the effect of RTD-1 in another animal model of RA, such as collagen induced arthritis, is likely to provide additional insights into anti-arthritic mechanisms mediated by θ-defensins.

Our data suggest that RTD-1 modulates arthritogenic pathways at more than one level. For example, down-regulation of inflammatory cytokine expression has been shown to occur at the transcriptional and post-transcriptional levels, the former revealed in studies on THP-1 macrophages wherein RTD-1 was shown to down-regulate MAP kinases and NF-ĸB activation, inhibiting transcription of TNFα, IL-1β, IL-8, CCL-3, and CCL-4 [15]. Post-translational regulation of arthritogenic mediators is exemplified by the rapid and potent inhibition of ADAM17 by RTD-1 which blocks TNFα release [39]. Consistent with this effect, RTD-1 treatment of PIA rats suppressed expression of joint IL-1β, a central mediator of arthritis which is upregulated by TNFα [35, 47]. RTD-1 also acts directly on FLS to reduce the production of IL-6 and reduces cell invasiveness, two processes involved in joint damage. Studies are underway to further delineate the pathways that mediate RTD-1 modulation of other inflammatory/arthritogenic cytokine networks implicated in RA.

The inhibitory action of RTD-1 on arthritogenic proteases, including MMPs and cathepsin K, suggests another anti-arthritic mechanism that may be operative *in vivo*. Results of the current study demonstrate that RTD-1 is a cross class anti-protease that inhibits zinc metalloproteinases (ADAMs and MMPs) and cysteine cathepsins. The inhibitory potency of RTD-1 against a panel of MMPs and cathepsins varies substantially, but plasma levels (C_max_) achieved following s.c. treatment approximate or exceed the IC_50_s of ADAM17 and cathepsin K, potentially providing a rapid mechanism for regulating these arthritogenic proteases, in the former case down-regulating local TNFα levels and downstream effects (e.g., IL-1β, IL-6, MMP expression). This may also contribute to inhibition of FLS invasiveness mediated by low concentrations of RTD-1.

θ-defensins are expressed at high levels in leukocytes and other tissues of Old World monkeys [8, 11–13]. However, θ-defensins are absent in hominids as a result of pseudogenization of θ-defensin precursor genes during primate evolution [48]. Evidence suggests that the expression of θ-defensins in rhesus macaque neutrophils underlies the superior antimicrobial properties of azurophil granules from these cells, compared to those of humans [11]. Further, θ-defensins possess unique immunomodulatory properties lacking in human α- and β-defensins [9, 15], potentially contributing to the marked resistance of θ-defensin expressing monkeys (e.g., macaques, vervets, and baboons) to endotoxin [49].

The results of this study indicate that θ-defensin-like cyclic peptides have potential as new agents for treatment of RA. RTD-1 itself is a pharmaceutical candidate as this naturally occurring molecule is well tolerated when administered subcutaneously (this study and ref. [9]), and by intravenous, intramuscular, and intraperitoneal routes (our unpublished results). RTD-1 was non-immunogenic (Fig 5), circumventing the development of neutralizing antibodies, a problem encountered by a subset of patients in response to biologic therapeutics [50]. Further, RTD-1 is highly stable in biological matrices [9], has an unusually long terminal half-life allowing for infrequent dosing (Fig 4), and shows no evidence of being immuno-compromising [9, 14]. The concept of using a nonhuman primate peptide represents a novel *retroevolutionary* therapeutic paradigm whereby products of genes expressed in Old World monkeys may be used to treat a human autoimmune disorder.

## Supporting information

S1 Fig PIA histology. Additional examples of joint histology of naïve, PIA, and RTD-1-treated PIA rats from which representative micrographs were selected in Fig 2C. Histology images were captured at either 10 or 4X magnification (organized by column), the top of each column contains a scale bar (10X = 500 μm, 4X = 1000 μm). The disease severity score of each individual sectioned limb is indicated as AS with possible scores of 0–4.
(TIF)

S1 Video RTD-1 arrests PIA and protects limb function. Scene one shows a healthy DA rat before PIA induction. Scene two is a PIA DA rat treated with saline for 11 days after disease presentation. Scene three is a PIA DA rat treated with RTD-1 (5 mg/kg s.c. qd x 11 days) for 11 days after disease presentation.
(MOV)

S2 Video RTD-1 treatment induces resolution of severe PIA and restores limb function. Scene one shows a healthy DA rat before PIA induction. Scene two is a PIA rat treated qd with saline 11 days after disease presentation. Scene three is the same rat in scene 2 after 8 days of RTD-1 treatment (5 mg/kg s.c. qd x 8 days).
(MOV)

S1 File NC3Rs ARRIVE guidelines checklist. (PDF)
