# Supplementary figures and images for: Suppression and resolution of autoimmune arthritis by rhesus θ-defensin-1, an immunomodulatory macrocyclic peptide
Source: PLoS One. 2017 Nov 16;12(11):e0187868. doi: 10.1371/journal.pone.0187868 (PMC5690597; doi:10.1371/journal.pone.0187868)

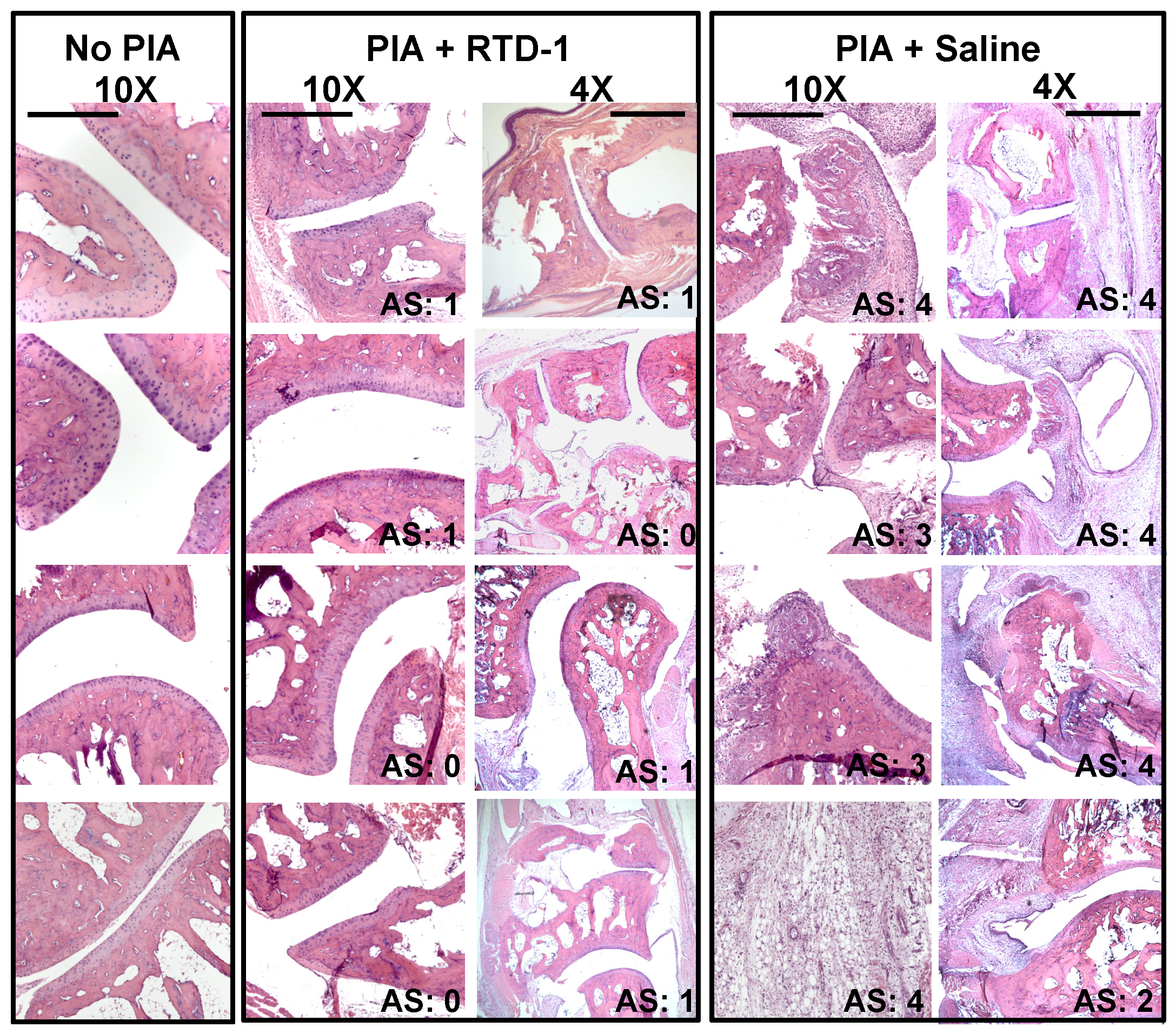

Supplement: S1 Fig — Additional examples of joint histology of naïve, PIA, and RTD-1-treated PIA rats from which representative micrographs were selected in Fig 2C. Histology images were captured at either 10 or 4X magnification (organized by column), the top of each column contains a scale bar (10X = 500 μm, 4X = 1000 μm). The disease severity score of each individual sectioned limb is indicated as AS with possible scores of 0–4. (TIF) [file pone.0187868.s001.tif]
